# Ophthalmic services in Shanghai 2017: a cataract-centric city-wide government survey

**DOI:** 10.1186/s12913-021-07048-1

**Published:** 2021-10-02

**Authors:** Xiangjia Zhu, Yu Du, Wenwen He, Jinhui Dai, Minjie Chen, Peijun Yao, Han Chen, Hui Ren, Yuan Fang, Shensheng Tan, Yi Lu

**Affiliations:** 1 Eye Institute of Eye and Ear, Nose, and Throat Hospital of Fudan University; Key Laboratory of Myopia, Ministry of Health; Key Laboratory of Visual Impairment and Restoration of Shanghai, Fudan University; Key NHC key Laboratory of Myopia, Fudan University; Chinese Academy of Medical Sciences, 83 Fenyang Road, Shanghai, 200031 China; 2 Shanghai Medical Quality Control Management Center, 1477 West Beijing Road, Shanghai, 200040 China

**Keywords:** Ophthalmic service, Cataract surgery, Public hospital, Private hospital, China

## Abstract

**Background:**

Demand for eye care has increased in recent decades in China due to rapid socioeconomic development and demographic shift. Knowledge of output and productivity of ophthalmic services would allow policymakers to optimize resource allocation, and is therefore essential. This study sought to map the landscape of ophthalmic services available in Shanghai, China.

**Methods:**

In 2018, a government-led survey was conducted of all 86 tertiary/secondary hospitals and five major private hospitals providing eye care in Shanghai in the form of electronic questionnaire, which encompassed ophthalmic services (outpatient and emergency room [ER] visit, inpatient admissions, and surgical volume) and service productivity in terms of annual outpatient and ER visits per doctor, inpatient admissions per bed, and surgical volume per doctor. Comparisons were made among different levels of hospitals with categorical variables tested by Chi-square analysis.

**Results:**

The response rate was 85.7%. The Eye and Ear, Nose, and Throat (EENT) Hospital was the largest tertiary specialty hospital, and alone contributed to the highest 21.0% of annual ophthalmic outpatient and ER visits (visits per doctor: 5460), compared with other 26 tertiary hospitals, 46 secondary hospitals and five private hospitals (visits per doctor: 3683, 4651 and 1876). The annual inpatient admission was 20,103, 56,992, 14,090, and 52,047 for the EENT Hospital, all the other tertiary hospitals, secondary hospitals and five private hospitals, respectively. Turnover rates were highest for the EENT Hospital and private hospitals. The average surgical volume at the EENT Hospital was 72,666, exceeding that of private (15,874.8) and other tertiary hospitals (3366.7). The EENT Hospital and private hospitals performed 16,982 (14.2%) and 55,538 (46.6%) of all cataract surgeries. Proportions of both complicated cataractous cases and complicated cataract surgeries at the EENT Hospital was the highest, followed by other tertiary and secondary/private hospitals (*P* < 0.0001).

**Conclusions:**

In Shanghai, public providers dominate ophthalmic services especially for complicated cases, with almost one fifth of services provided by the EENT Hospital alone, while private sectors, though not large in number, still effectively help meet large proportions of eye care demand. Optimization of hierarchical medical system is warranted to improve the efficiency and standardization of ophthalmic services.

**Supplementary Information:**

The online version contains supplementary material available at 10.1186/s12913-021-07048-1.

## Background

China, as the most populous country in the world, has achieved remarkable progress in transitioning from a centrally planned economy towards a market-oriented economy over the last 40 years. However, the efficiency and equity of China’s healthcare system, which potentially affects the lives of nearly 1.40 billion people, have developed unsatisfactorily [1, 2]. With the rapid demographic shift towards an aging population and the simultaneous epidemiological change of the disease burden into predominantly non-communicable diseases, especially age-related diseases, it is imperative for China to revamp the existing healthcare delivery system to meet the increasing demand for medical services [3].

Despite rapid development, public hospitals are struggling to cater for the rising numbers of patients. Therefore, the government has implemented numerous favorable policies to facilitate the growth of private services to expand access to healthcare and to alleviate the burden of healthcare delivery [4, 5]. In 1980, China first issued a policy that encouraged qualified medical professionals to engage in private practice [6]. In 2010, the State Council published a policy document that further encouraged social capital investment in healthcare [7]. By 2017, the number of private hospitals in China had increased to 447,160 [8], and these hospitals address the shortage of public healthcare services. Nevertheless, careful attention should be given to the efficiency of hospitals to evaluate their performance and to improve the coordination of different sectors.

Impaired vision is a high-priority public health issue, and population aging in China is causing a dramatic increase in the burden of age-related ocular diseases, such as cataracts and macular degeneration [9–11]. To improve eye care services in China, data are needed on the current availability and use of ophthalmic services. Shanghai is the economic center and the second most populous city in China, and faces tough challenges associated with an aging society. In 2016, 13.0% of the registered population was over 65 years old, a percentage that increased to 21.8% in 2017 according to data provided by the Shanghai Municipal Statistics Bureau.

Considering these data, the future demand for eye care services is likely to outpace the available resources. Thus, periodic assessment of the availability and productivity of ophthalmic services in Shanghai during the current period of healthcare reform could be very meaningful. However, few studies have addressed this issue to date.

In 2018, the Shanghai Quality Control Center of Clinical Ophthalmology, an evidence-based center that conducts clinical care evaluations of ophthalmology, launched the first electronic questionnaire survey to obtain baseline information about the characteristics of eye care providers and their practices in Shanghai. The survey investigated the distribution of the ophthalmic services across different subspecialties. It paid particular attention to cataract surgery because it was classified as the prioritized eye disease to be addressed in China in the 13th 5-Year National Plan of Eye Health launched by the National Health Commission in 2016.

## Methods

This was an observational, cross-sectional, questionnaire survey of hospitals providing specialty eye care in Shanghai. This study involved the analysis of data acquired from a government-led questionnaire survey, which did not involve any human participants, personal identifiable information or animal experiments. Thus, according to the Ethics Committee at the EENT Hospital of Fudan University, this study involved no ethical issues, and was reviewed and deemed exempt from formal ethics approval or consent forms from the participating hospitals. Via emails or telephone calls with the administrative offices at the target hospitals, we explained the purpose of the survey and asked for their participation. Only those who agreed to participate completed the questionnaire.

### Participating hospitals

We considered it essential to collect data from all 86 tertiary and secondary hospitals with an ophthalmology department and from five major high-volume private eye hospitals.

### Questionnaire

The questionnaire (Supplementary file) was developed by a panel of eye doctors in Shanghai based on previous published reports of other countries and was sent to the eligible hospitals in 2018 to collect information of ophthalmic services for the 2017 fiscal year. Data collection was completed in October 2018. The questionnaire had two main parts: the basic characteristics of the hospital and the level of ophthalmic specialty services, which included (1) outpatient and inpatient services, (2) professional staff, (3) ophthalmic equipment, (4) volumes of different types of ophthalmic procedures, and (5) specific data on cataract surgery. Since the questionnaire used in this survey only included data collection of objective subjects without any subjective judgement, validation was not necessary.

The questionnaires were sent by standard mail to all eligible hospitals by the Shanghai Quality-control Center of Clinical Ophthalmology. The correspondence included a clear explanation of the survey’s purpose and the questionnaires were to be completed by an appropriate person in the administrative office at each hospital to ensure accuracy. Deadline was October 2018 with a reminder email sent to the hospitals if they did not return the questionnaire.

### Measures and analysis

The responses to the questions were summarized as proportions or means. Hospital output was assessed in terms of the number of outpatient and emergency room (ER) visits, the number of the inpatient admissions, and surgical volume. Service efficiency was measured as the annual outpatient and ER visits per doctor, annual inpatient admissions per bed, and the annual surgical volume per doctor. Information pertaining to the demographic data of Shanghai in 2017 was extracted from China’s Health Statistics Yearbook 2018. Comparisons among categorical variables among different levels of hospital were conducted by Chi-square analysis (Prism Version 9.9.1, GraphPad Software, LLC).

Among the tertiary hospitals surveyed in this study, one stood out, the EENT Hospital of Fudan University, as the largest hospital specialized in ophthalmology and otolaryngology in China. The EENT Hospital treats a much greater number of ophthalmic patients than other hospitals in Shanghai. Therefore, its data were analyzed separately from those of other tertiary hospitals.

## Results

### Participating hospitals

Among 91 eligible hospitals, 78 hospitals responded (response rate of 85.7%), of which 27 were tertiary hospitals (EENT Hospital plus 26 other tertiary hospitals), 46 were secondary hospitals, and five were major high-volume private hospitals. All the tertiary hospitals and private hospitals responded while 13 secondary hospitals did not.

### Basic characteristics of the hospitals: professionals, hospital beds, and equipment

In 2017, there were over 3000 doctors, nurses and technicians providing eye care in Shanghai, representing approximately 0.49 ophthalmologists per 10,000 population. Overall, 83.7% of eye doctors worked in public hospitals, which included 14.8% at the EENT Hospital and 47.8% at the other 26 tertiary hospitals (Table 1). The mean number of eye doctors with senior titles (chief physician and associate chief physician) was greatest at the EENT Hospital (67), followed by private hospitals (19.6), other tertiary hospitals (8.0), and secondary hospitals (2.0).

**Table 1:** Total number and composition of professional and allied staff at each hospital level

Staff Number	Chief Physician	Associate Chief Physician	Attending Physician	Resident	Ophthalmic Nurse	Theater Nurse	Ordinary Nurse	Optometrist	Total
EENT Hospital	28	39	39	70	145	109	36	20	486
Other tertiary hospitals	95 (3.7)	113 (4.3)	150 (5.8)	209 (8.0)	275 (10.6)	97 (3.7)	178 (6.8)	112 (4.3)	1229 (47.3)
Secondary hospitals	44 (1.0)	47 (1.0)	115 (2.5)	44 (1.0)	131 (2.8)	56 (1.2)	75 (1.6)	41 (0.9)	553 (12.0)
Private hospitals	57 (11.4)	41 (8.2)	43 (8.6)	52 (10.4)	263 (52.6)	56 (11.2)	207 (41.4)	41 (8.2)	760 (152.0)
Total	224	240	347	375	814	318	496	214	3028

Numbers in brackets represent the mean number of staff at each hospital level.

*EENT Hospital* Eye and Ear, Nose, and Throat Hospital of Fudan University

The survey recorded 1492 beds for hospitalized ophthalmic patients in Shanghai in 2017. Of these, 10.1, 50.7, and 16.2% were at the EENT Hospital, other tertiary hospitals, and secondary hospitals, while the five private hospitals had 23.1% of beds. On average, the number of beds at the EENT Hospital (150) exceeded the mean number of beds at private hospitals (68.8), tertiary hospitals (29.1), and secondary hospitals (5.2).

In terms of the distribution of medical personnel among hospitals, the EENT Hospital was the best equipped, with the most comprehensive ophthalmic examinations available, followed by private hospitals (Table 2). Unsurprisingly, the secondary hospitals lacked complex or advanced equipment, such as fluorescence angiography, confocal microscopy, vitrectomy instruments, and femtosecond lasers, compared with tertiary hospitals.

**Table 2:** Availability of ophthalmic equipment at each hospital level

Ophthalmic Equipment		Coverage Rate (%)			
		Public			Private hospitals
		EENT Hospital	Other tertiary hospitals	Secondary hospitals	
Slit lamp		100	100	100	100
Direct ophthalmoscopy		100	100	95.7	100
Indirect ophthalmoscopy		100	76.9	15.2	60
Tonometer	Applanation	100	34.6	4.3	20
	Schiotz	100	15.4	21.7	20
	NCT	100	100	97.8	100
	Tonopen	100	11.5	0	40
Perimeter	Static	100	73.1	50	100
	Goldman	100	19.2	10.9	80
Autorefractor		100	88.5	89.1	100
Fundus camera		100	84.6	84.8	100
Nd Yag laser machine		100	73.1	39.1	80
Laser treatment machine of fundus		100	88.5	47.8	100
Biometry	A-ultrasound	100	76.9	71.7	100
	IOLmaster	100	69.2	34.8	100
	B-ultrasound	100	76.9	73.9	100
	UBM	100	53.8	13	100
	Lenstar	100	30.8	4.3	0
Ophthalmic operating microscope		100	100	67.4	100
Fluorescence angiography equipment		100	69.2	47.8	100
ICG angiography equipment		100	30.8	13	20
Corneal topographer		100	61.5	23.9	80
OCT	Anterior-segment	100	42.3	26.1	50
	Glaucoma	100	38.5	21.7	80
	Fundus	100	84.6	71.7	100
Visual electrophysiology examination machine		100	61.5	15.2	100
Specular microscope of cornea		100	76.9	47.8	100
Confocal microscope		100	15.4	2.2	20
Wavefront aberrometer		100	26.9	0	20
Contrast sensitivity device		100	26.9	2.2	50
Corneal thickness measuring device		100	46.2	26.1	20
Lens box		100	96.2	84.8	100
Comprehensive refractometer		100	88.5	67.4	100
Quick sterilizer		100	61.5	39.1	100
Excimer laser instrument		100	26.9	2.2	100
Femtosecond laser equipment	For refractive surgery	100	23.1	0	100
	For cataract surgery	100	3.8	0	80
Phacoemulsification instrument		100	80.8	67.4	100
Vitrectomy instrument		100	61.5	28.3	100

*EENT Hospital* Eye and Ear, Nose, and Throat Hospital of Fudan University, *NCT* non-contact tonometer, *Nd Yag* neodymium-doped yttrium aluminum garnet, *UBM* ultrasound biomicroscope, *ICG* indocyanine green, *OCT* optical coherence tomography

### Ophthalmic service output and productivity

The output of ophthalmic services was evaluated in terms of the number of outpatient and ER visits, the number of inpatient admissions, and surgical volume.

The annual number of ophthalmic outpatient and ER visits in 2017 was 4,574,143, of which 21.0% were provided by the EENT Hospital alone and 45.7% by other tertiary hospitals (Table 3). Private hospitals accounted for just 7.9% of the total number, which was relatively low considering they accounted for 16.3% of the total number of eye doctors. Generally, 31.7% of outpatient and ER visits in Shanghai were due to non-local patients. This percentage was the highest at the EENT Hospital (61.0%) followed by other tertiary hospitals (30.6%), whereas secondary and private hospitals mostly served local patients (over 80%) (*P* < 0.0001, Chi-square analysis). Regarding the productivity of outpatient services, the number of outpatient and ER visits per eye doctor varied considerably among hospitals: 5460 at the EENT Hospital, 3683 at other tertiary hospitals, and only 1876 at private hospitals.

**Table 3:** Ophthalmic services provided at each hospital level

Output of ophthalmic services	EENT Hospital	Other tertiary hospitals	Secondary hospitals	Private hospitals
**Outpatient and emergency room visit**				
Local	374,768	1,449,000	984,600	315,700
Non-local	586,175	639,400	178,200	46,300
Total	960,943 (21.0%)	2,088,400 (45.7%)	1,162,800 (25.4%)	362,000 (7.9%)
Average visit	960,943	80,323.1	25,278.3	72,400.0
**Inpatient admission**				
Total inpatients	20,103 (14.0%)	56,992 (39.8%)	14,090 (9.8%)	52,047 (36.3%)
Average inpatients	20,103	2192	306.3	10,409.4
**Surgical volumes**				
Total volumes	72,666 (27.4%)	87,533 (33.0%)	25,792 (9.7%)	79,374 (29.9%)
Average volumes	72,666	3366.7	560.7	15,874.8

The numbers in brackets represent the percentage of the total number for all hospitals

*EENT Hospital* Eye and Ear, Nose, and Throat Hospital of Fudan University

The total number of ophthalmic inpatient admissions in Shanghai was 143,232, of which 14% were at the EENT Hospital, 39.8% at other tertiary hospitals, and 36.3% at private hospitals (Table 3). The turnover rate, measured as the annual inpatient admissions per bed, was higher at the EENT Hospital and private hospitals (134 and 151, respectively) than at other tertiary hospitals and secondary hospitals (75 and 58, respectively).

Ophthalmic procedures are another major aspect of eye care provided to the public. A total of 265,365 ophthalmic procedures were performed in 2017. The EENT Hospital, other tertiary hospitals, and the private hospitals each provided about 30% of the total surgical volume (Table 3). On average, the EENT Hospital was ranked first in terms of the annual surgical volume, having performed 72,666 procedures, which greatly exceeded that of private hospitals (15,874.8) and other tertiary hospitals (3366.7). The annual surgical volume per doctor was also greatest at the EENT Hospital (412.9), followed by private hospitals (411.3) and other tertiary hospitals (154.4).

**Fig. 1:**
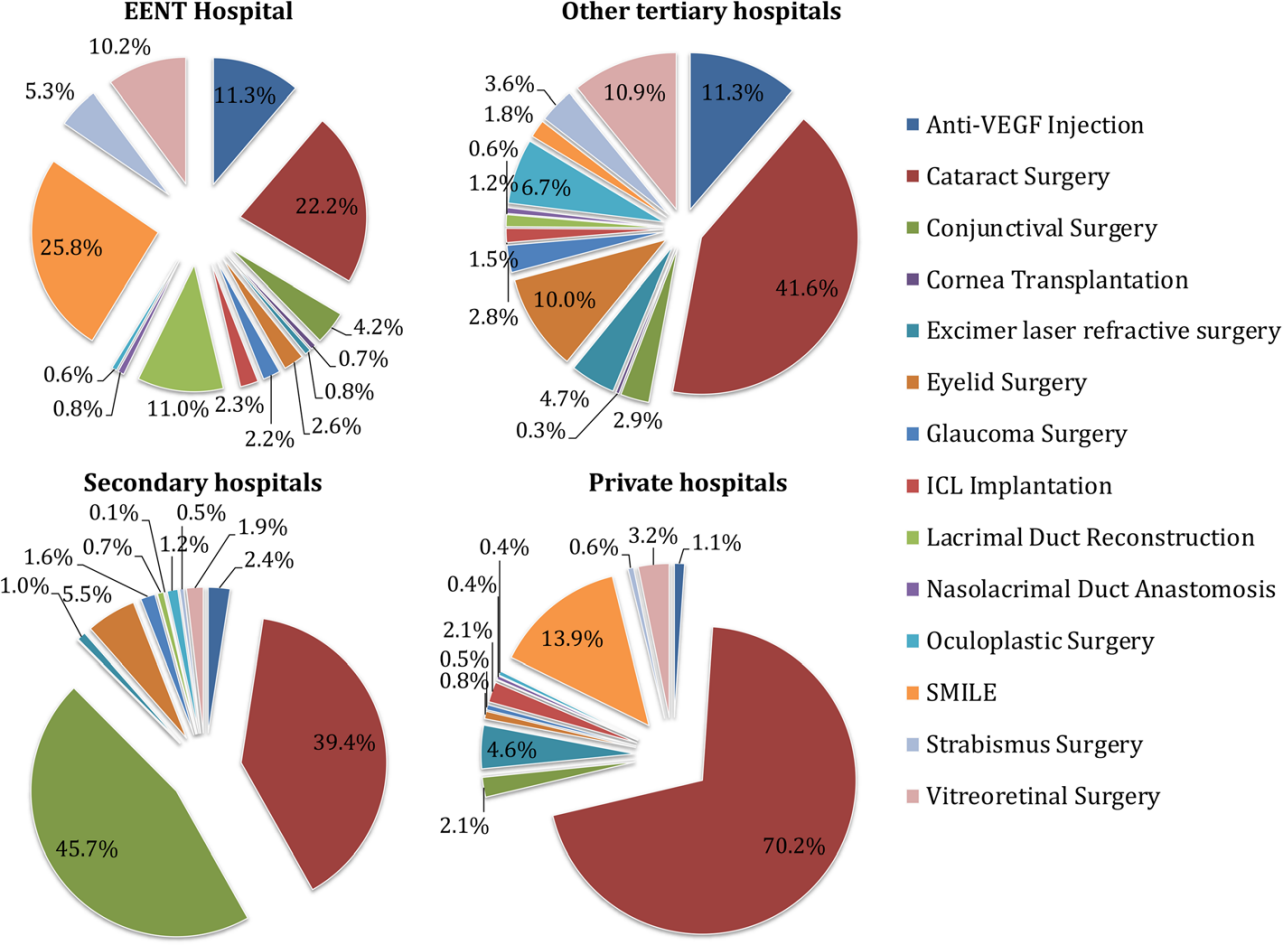
Comparison of the surgical volume for different subspecialties at each hospital level. EENT Hospital: Eye and Ear, Nose, and Throat Hospital of Fudan University; ICL: implantable collamer lens; SMILE: small incision lenticule extraction

In terms of the type of ophthalmic procedures, differences among procedure compositions of EENT Hospital, other tertiary hospitals, secondary hospitals, and private hospitals were significant (*P* < 0.0001, Chi-square analysis). Generally, cataract surgery was the most common (44.3%), followed by small incision lenticule extraction (SMILE, 12.0%) and vitreoretinal surgery (7.6%). At the EENT Hospital, SMILE was the most frequent procedure (25.8%), followed by cataract surgery (22.2%), anti-vascular endothelial growth factor injection (11.3%), lacrimal duct reconstruction (11.0%) and vitreoretinal surgery (10.2%). At private hospitals, cataract surgery accounted for 70.2% of procedures and SMILE accounted for 13.9% (Fig. 1). At secondary hospitals, the percentage of extraocular procedures, particularly conjunctival surgery such as pterygium surgery (45.7%), was much greater than that at tertiary hospitals and private hospitals. Complicated eye surgeries, such as vitreoretinal surgery and corneal transplantation, were mostly performed in tertiary hospitals. Private hospitals performed just 12.2% of these procedures, even though they had sufficient senior doctors, adequate hospital beds, and comprehensive equipment.

### Cataract surgery service

In 2017, totally 119,264 cataract surgeries were performed in Shanghai, of which EENT Hospital alone contributed 14.2%, the other 26 tertiary hospitals contributed 30.7%, 46 secondary hospitals contributed 8.5% and private hospitals contributed 46.6% (Table 4). Based on the data provided by Shanghai Municipal Bureau of Statistics, there were around 24.18 million residents in Shanghai in 2017. The cataract surgical rate (cataract operations done per million populations per year) might be around 4932.3 (119,264/ 24.18) in 2017 in Shanghai. However, given that the total number of cataract surgeries included a certain proportion of surgeries performed on non-residents, this number was higher than the 4251 reported by the National Cataract Surgery Information Reporting System.

**Table 4:** Cataract surgical procedures provided at each hospital level

Data on cataract surgery	EENT Hospital	Other tertiary hospitals	Secondary hospitals	Private hospitals	Total
**Cataract type**					
Age-related cataract	7132 (42.0%)	20,747 (56.6%)	6959 (68.8%)	37,949 (68.3%)	72,787 (61.0%)
Highly myopic cataract	5604 (33.0%)	8494 (23.2%)	1346 (13.3%)	9654 (17.4%)	25,098 (21.0%)
Diabetic cataract	2377 (14.0%)	5362 (14.6%)	1428 (14.1%)	7214 (13.0%)	16,381 (13.7%)
Traumatic cataract	672 (4.0%)	938 (2.6%)	66 (0.7%)	29 (0.1%)	1705 (1.4%)
Congenial cataract	664 (3.9%)	845 (2.3%)	67 (0.7%)	28 (0.1%)	1604 (1.3%)
Others	533 (3.1%)	244 (0.7%)	248 (2.5%)	664 (1.2%)	1689 (1.4%)
Total	16,982 (14.2%)	36,630 (30.7%)	10,114 (8.5%)	55,538 (46.6%)	119,264
**Surgical techniques used in cataract surgery**					
Standard phacoemulsification	15,425 (90.8%)	35,019 (95.6%)	9840 (97.3%)	53,216 (95.8%)	113,500 (95.2%)
FLACS	251 (1.5%)	28 (0.1%)	0 (0.0%)	1358 (2.4%)	1637 (1.4%)
Extracapsular cataract extraction	130 (0.8)	334 (0.9%)	238 (2.4%)	316 (0.6%)	1018 (0.9%)
Intracapsular cataract extraction	15 (0.1%)	19 (0.1%)	0 (0.0%)	2 (0.0%)	36 (0.0%)
Scleral fixation of IOL	325 (1.9%)	305 (0.8%)	36 (0.4%)	320 (0.6%)	986 (0.8%)
Others	836 (4.9%)	925 (2.5%)	0 (0.0%)	326 (0.6%)	2087 (1.7%)
Total	16,982 (14.2%)	36,630 (30.7%)	10,114 (8.5%)	55,538 (46.6%)	119,264
**IOL used in cataract surgery**					
Monofocal IOL	15,866 (97.6%)	35,023 (98.1%)	9891 (99.9%)	53,322 (96.7%)	114,102 (97.5%)
Multifocal IOL	247 (1.5%)	379 (1.1%)	11 (0.1%)	1064 (1.9%)	1701 (1.5%)
Toric IOL	139 (0.9%)	276 (0.8%)	1 (0.0%)	722 (1.3%)	1138 (1.0%)
Toric multifocal IOL	0 (0.0%)	19 (0.1%)	0 (0.0%)	32 (0.1%)	51 (0.0%)
Total	16,252 (13.9%)	35,697 (30.5%)	9903 (8.5%)	55,140 (47.1%)	116,992
**CTR used in cataract surgery**					
CTR	228 (51.7%)	93 (76.2%)	1 (100.0%)	26 (83.9%)	348 (58.5%)
Modified CTR	213 (48.3%)	29 (23.8%)	0 (0.0%)	5 (16.1%)	247 (41.5%)
Total	441 (74.1%)	122 (20.5%)	1 (0.2%)	31 (5.2%)	595

The numbers in brackets represent the percentage of the total number of procedures performed at each hospital level. The numbers in brackets in the total row represent the percentage of the total number of procedures performed at all hospitals

*EENT Hospital* Eye and Ear, Nose, and Throat Hospital of Fudan University, *FLACS* femtosecond laser-assisted cataract surgery, *IOL* intraocular lens, *CTR* capsular tension ring

At the EENT Hospital, only 43.9% of cataract procedures were performed as inpatient surgery. This value increased to 76.9 and 80.3% at secondary and private hospitals, respectively. The mean wait time for cataract surgery ranged from 0.3 to 8 weeks. The daily number of cataract surgical procedures ranged between 20 and 80 at private hospitals, 68 at the EENT Hospital, and 20–65 at other tertiary hospitals. On average, the annual surgical volume per cataract surgeon was greatest at private hospitals (2136.1), followed by the EENT hospital (1306.3), and other tertiary hospitals (359.1).

Patient selection for cataract surgery showed clear trends among hospitals. The proportion of complicated cataractous patients varied significantly among EENT Hospital, other tertiary hospitals, secondary hospitals, and private hospitals (*P* < 0.0001, Chi-square analysis). At the EENT Hospital, 58.0% of cataract procedures were for complicated cataracts such as highly myopic cataract, traumatic cataract, and congenital cataract, requiring multi-subspecialty eye care, significantly higher than other hospitals on an average level. By comparison, this percentage was only 31.7% at private hospitals despite the large volume of cataract surgery (Table 4).

Regarding surgical techniques, standard phacoemulsification was the predominant cataract surgical technique (mostly > 95%). Of all femtosecond laser-assisted cataract surgical procedures (FLACS), 83% (1358) and 15% (251) were performed at private hospitals and the EENT Hospital, respectively. Meanwhile, the EENT Hospital performed 33.0% (325) of more difficult cataract procedures, such as scleral fixation of an intraocular lens (IOL) (Table 4). The proportion of complicated cataract surgery in the EENT Hospital was the highest (7.7%), followed by other tertiary hospitals (4.3%), secondary hospitals (2.7%), and private hospitals (1.7%) (*P* < 0.0001, Chi-square analysis).

A greater number of advanced IOLs, such as toric IOLs and multifocal IOLs, were implanted at the EENT Hospital (386), followed by private hospitals (mean 363.6), accounting for 2.4 and 3.3% of all IOL implanted at these hospitals. However, capsular tension ring (CTR) and modified CTR implantation were predominantly used at the EENT Hospital (74.1%, Table 4), followed by other tertiary hospitals (20.5%) and private hospitals (5.2%).

## Discussion

This paper provides detailed information about eye care providers in Shanghai in 2017, with the aim to understand the availability and productivity of ophthalmic services and to provide a foundation for quality control of ophthalmic care in the future. No government-led electronic questionnaire survey of this kind, enrolling nearly all eye care hospitals in Shanghai, has been performed previously. The ophthalmic services were evaluated in terms of the basic characteristics of the hospitals, as well as their service output and efficiency. The survey had a particular emphasis on cataract surgery to investigate differences in its provision, independently of the disparity in productivity among different hospitals.

With the revitalization of privatization in China, the number of private healthcare providers has expanded greatly in recent years [12]. The creation of the China Pilot Free-Trade Zone (Shanghai) in late 2013 provided an effective platform for foreign direct investment into the healthcare sector. According to the Annual Report on the National Health Service in China 2017, published by the National Health Commission of the People’s Republic of China, the number of patient visits to private hospitals increased by 17.0% in 2017 versus that in 2016, as compared with 4.2% in public hospitals. Moreover, China’s Health Statistics Yearbook 2018 reported that by the end of 2017, private providers accounted for nearly 50% (447,160/986,649) of all healthcare institutions in China [8]. Shanghai, the most economically developed city in China, has attracted significant private investment in healthcare and has seen a remarkable increase in the number of private hospitals in recent years.

Our findings capture some typical features of the public and private ophthalmic services in Shanghai. Most hospitals offering ophthalmic services were still public, similar to the characteristics of other provinces of China, such as Guangdong [13]. This was possibly due to historical reasons. Before the implementation of supporting policy and the revitalization of privatization, there were hardly any standard private sectors in realm of healthcare. Thus, patients tend to trust more in the reliability of public hospitals. However, in terms of the basic characteristics of the hospitals, the private sector has now greatly developed to an extent comparable with that of public hospitals of the highest accreditation. Similar to the medical systems of other countries, the private clinics tended to optimize non-clinical factors, such as facilities and wait times, more than public providers [14]. Social investment in private hospitals allowed them to employ many reputable doctors, construct larger-scale hospitals, and procure up-to-date equipment to attract patients [13, 15, 16]. This may help explain why the five private hospitals in Shanghai were better equipped than most of the public hospitals in Shanghai. Nevertheless, the EENT Hospital was still the best equipped with the highest percentage of senior doctors, the most beds and highest availability of almost all commercially available equipment for ophthalmic examinations and procedures.

Although there were gaps in the objective characteristics between hospital levels, the disparity in productivity was more concerning. The productive efficiency of hospitals is crucial to their future development, as a guarantee of their potential to meet the ever-increasing demand for healthcare. We found that 20.7% of the annual outpatient and ER visits were served by 14.8% of eye doctors at the EENT Hospital. In contrast, private hospitals contributed to just 7.8% of the total volume, despite employing 16.2% of the registered workforce, indicating an obvious disparity in technical efficiency. Similar findings were identified in terms of inpatient admissions and surgical volumes among the levels of hospitals. The turnover rate at the EENT Hospital was almost two to three times higher than that at other tertiary and secondary hospitals. Moreover, the EENT Hospital performed 27.4% of all ophthalmic procedures in Shanghai, whereas the average volume at other hospitals was less than a quarter of this number.

Among the five private hospitals, the surgical volume per doctor was very close to that at the EENT Hospital, which may suggest a much greater efficiency at private hospitals than at most public hospitals. Nevertheless, the difficulty levels of eye care differed markedly among these hospitals. At the EENT Hospital, vitreoretinal surgery, one of the most complicated ophthalmic procedures, accounted for 10.2% of its surgical volume. A similar value of 10.9% was found at other tertiary hospitals, but this dropped to 1.9% at secondary hospitals, due to their lower technical level. Meanwhile, this value was also low, 3.2%, at private hospitals despite their extensive staff and facilities.

Private hospitals performed 29.9% of the total surgical volume in Shanghai, but 70.2% of this was due to cataract surgery. One possible explanation is that the accumulated experience at tertiary hospitals, and greater support from local government and affiliating universities, mean that their professional capabilities had reached a level similar to that in most developed countries [17], and they were able to provide comprehensive eye care across multiple subspecialties. However, unlike the social objectives of public hospitals, the private hospitals tended to provide services with a high profit margin. Cataract surgery and refractive surgery, two highly cost-effective ophthalmic procedures, have a huge potential market considering cataract and refractive error are the two major causes of visual impairment worldwide.

The types of cataract procedures also demonstrate the clear distinction between high-volume public hospitals and private hospitals. Advanced cataract techniques, including FLACS and implantation of advanced IOLs, were mostly performed at the EENT Hospital and private hospitals. However, private hospitals tended to perform fewer procedures in patients with complicated cataracts and performed fewer complex surgical techniques such as scleral fixation or implantation of CTR. Despite this, the private hospitals still provide an important aspect of eye care in Shanghai. The five major high-volume private hospitals performed over 46% of all cataract surgeries in 2017 in Shanghai, and contributed to the increase in the number of cataract surgeries from 2313 in 2012 to 4251 in 2017. Accordingly, private hospitals helped alleviate the imbalance between medical supply and demand, accelerating the achievement of universal coverage planned in China’s new round of healthcare reform. Nevertheless, appropriate supervision and management by the government is necessary to ensure standardization of healthcare services and to reduce the profit-driven focus in healthcare.

Collectively, it is not difficult to perceive the high output and productivity of the EENT Hospital for providing comprehensive eye care to people in Shanghai. The EENT Hospital, alone, provided nearly one fifth of eye care services in Shanghai. It also treated a huge number of non-local patients. However, this high productivity could be a double-edged sword as it allowed easier access to high-quality ophthalmic care to more patients, but this overload may overburden the doctors and impair the quality of the service they provide [18].

The high productivity at a single hospital may be due to a combination of internal and external factors. Owing to its large scale, professional staffing and the most comprehensive equipment, the EENT Hospital is ranked second nationally in the field of ophthalmology. These features mean it attracts many patients from neighboring provinces, or further field in China [19]. Another explanation may relate to an ineffective referral triage process. Unlike the process of seeing a doctor in an established medical system, such as in the United States, patients in China do not need to obtain a referral from a family doctor and can bypass the primary healthcare system. Patients flocking to large tertiary hospitals, whether necessary or not, forced the passive increase in efficiency at these hospitals. The over-influx of patients with minor ailments, which could be treated in primary or secondary hospitals, led to the aggravation of the so-called “three long, one short” problem at these large hospitals [1], comprising long registration times, long wait times, long payment queue times, and short visit times, resulting in the pseudo-proposition of difficult access to health services. Overall, a deficit of ophthalmologists remained in Shanghai, with a density of only 0.49 ophthalmologists per 10,000 population, far below the values of 2.56 in Greece, 1.93 in Switzerland in 2017, and 0.60 in United States in 2014.

Nevertheless, the wait time for a consultation in China was relatively short. In Toronto, patients undergoing diagnostic testing for uveitis waited > 40 days [20]. In China, some patients with serious diseases might benefit from the current system, but the medical resources in tertiary hospitals were exhausted, and largely wasted at lower-level hospitals. In the long term, only an effective referral triage process could address the seemingly uneven distribution of medical resources.

## Conclusion

We performed a city-wide questionnaire survey that provides an overview of the eye care services available in Shanghai. Notwithstanding the rapid development of private sectors, public hospitals still provide the bulk of ophthalmic services. In the hierarchic public system, the EENT Hospital provided almost one fifth of eye care services in Shanghai. The city’s services were largely supplemented by private hospitals, which help meet the growing medical demands for treating common diseases. However, further optimization of the hierarchic medical system with proper policy guidance is still necessary to standardize and improve the efficiency of current ophthalmic services.

## Supplementary Information

**Additional file 1.**

## Data Availability

The data are available from the authors upon reasonable request and with permission from the Shanghai Medical Quality Control Management Center.

## Abbreviations

ER: Emergency room
EENT: Eye and Ear, Nose, and Throat
SMILE: Small incision lenticule extraction
FLACS: Femtosecond laser-assisted cataract surgery
IOL: Intraocular lens
CTR: Capsular tension ring
