# Supplementary material for: Ophthalmic services in Shanghai 2017: a cataract-centric city-wide government survey
Source: BMC Health Serv Res. 2021 Oct 2;21:1043. doi: 10.1186/s12913-021-07048-1 (PMC8487503; doi:10.1186/s12913-021-07048-1)
Supplement: Supplementary file 1 — Additional file 1. [file 12913_2021_7048_MOESM1_ESM.pdf]

Questionnaire on Current Status of Ophthalmic Services in Shanghai

Hospital:

Please contact shykzk@126.com with any questions about this questionnaire, or if you require further assitance.

I. GENERAL CONDITION

Address:						
Tel:			Postal code：		Fax：	
Director of the depatment of Ophthalmology：			Tel：		E-mail：	
Contact：			Tel：			
Types of the healthcare facility:	1 = Public hospital	I = Primary				
		II = Secondary				
		III = Tertiary				
	2 = Private hospital					

II. LEVEL OF OPHTHALMIC SPECIALTY SERVICE

**Outpatient and inpatient service**				
Total outpatient and emergency department visits in 2017				
	Local patients		Non-localt patients	
Number of hospital beds for ophthalmic patients			Amount of inpatient admission	

**Professional staff and allied personnel**							
Number of chief physician		Number of associate chief physician		Number of attending doctor		Number of resident	
Number of ophthalmic nurse		Number of theater nurse		Number of ordinary nurse in department of Ophthalmology		Number of optometrist	

**Ophthalmology Equipment**		0=No<br>1=Yes
Slit lamp		
Direct ophthalmoscopy		
Indirect ophthalmoscopy		
Tonometer	Applanation	
	Schiotz	
	NCT	
	Tonopen	
Perimeter	Static	
	Goldman	
Autorefractor		
Fundus camera		
Nd Yag laser machine		
Laser treatment machine of fundus		
Biometry	A-ultrasound	
	IOLmaster	
	B-ultrasound	
	UBM	
	Lenstar	
Ophthalmic operating microscope		
Fluorescence angiography equipment		
ICG angiography equipment		
Corneal topographer		
OCT	Anterior-segment	
	Glaucoma	
	Fundus	
Visual electrophysiology examination machine		
Specular microscope of cornea		
Confocal microscope		
Wavefront aberrometer		
Contrast sensitivity device		
Corneal thickness measuring device		
Lens box		
Comprehensive refractometer		
Quick sterilizer		
Excimer laser instrument		
Femtosecond laser equipment	For refractive surgery	
	For cataract surgery	
Phacoemulsification instrument		
Vitrectomy instrument		

**Surgical volumes**					
Total surgical volumes in 2017		Outpatient and emergency surgery		Inpatient surgery	
Cataract surgery		Cornea transplantation		Glaucoma surgery	
Anti-VEGF injection		Vitreoretinal surgery		Strabismus surgery	
Oculoplastic Surgery		Eyelid surgery		Conjunctival surgery	
SMILE		Excimer laser refractive surgery		ICL implantation	
Lacrimal Duct Reconstruction		Nasolacrimal Duct Anastomosis			

**Data on Cataract Surgery**					
Amount of inpatient cataract surgery		Amount of outpatient cataract surgery		Waiting time for cataract surgery (in weeks)	
Average amount of daily cataract surgery					
Amount of standard phacoemulsification		Number of implanted monofocal IOLs		Surgical volumes performed on age-related cataract	
Amount of femtosecond laser- assisted cataract surgery		Number of implanted multifocal IOLs		Surgical volumes performed on highly myopic cataract (axial length≥ 26mm)	
Amount of extracapsular cataract extraction		Number of implanted toric IOLs		Surgical volumes performed on diabetic cataract	
Amount of intracapsular cataract extraction		Number of implanted toric multifocal IOLs		Surgical volumes performed on congenital cataract	
Amount of scleral fixation of IOL		Number of implanted CTR		Surgical volumes performed on traumatic cataract	
Others		Number of implanted modified CTR		Surgical volumes performed on other complicated cataract	
